# Exploring the effects of outdoor physical work in the heat, with or without sodium bicarbonate supplementation, on markers of acute kidney injury

**DOI:** 10.14814/phy2.70472

**Published:** 2025-07-23

**Authors:** Jason Siegler, Brooke Butterick, Raul Freire, Jonathan Specht, Fabiano Amorim

**Affiliations:** ^1^ Integrative Human Performance Lab, College of Health Solutions Arizona State University Tempe Arizona USA; ^2^ Department of Health, Exercise & Sports Sciences University of New Mexico Albuquerque New Mexico USA

**Keywords:** heat stress, kidney injury, metabolic alkalosis, physical work

## Abstract

Acute kidney injury (AKI) has been observed after prolonged physical activity in the heat. Although a range of strategies to reduce the incidence of AKI have been investigated, sodium bicarbonate (SB) supplementation may mitigate metabolic stress during physical exertion and potentially alleviate renal workload by lowering glomerular filtration demand, reducing bicarbonate reabsorption, and preserving kidney microcirculation. Therefore, the primary objective of this study was to investigate the effect of SB ingestion on markers of AKI. Fourteen healthy men and women (*n* = 6) completed two (SB & placebo) experimental 2‐hr outdoor work sessions in the heat (~35°C; ~20% humidity) designed to simulate construction tasks. Changes in acid–base balance, markers of kidney injury (NGAL, TIMP2, IGFBP‐7), thermotolerance, and work achieved throughout the experimental trials were analyzed using a linear mixed model with two‐way repeated measures. Despite inducing a significant level of alkalosis in the SB trial (7.40 ± 0.03 vs. 7.45 ± 0.03; *p* < 0.001), no changes were observed in urinary NGAL, TIMP2, or IGFBP‐7 concentrations (*p* > 0.27). Core temperature and heart rate were elevated throughout the work session in the SB condition (mean increase of 0.2 ± 0.1°C and 9 ± 3 bpm; *p* < 0.025), but the rating of perceived effort was lower when compared to placebo (0.3 ± 0.1 au; *p* = 0.003). Although the environmental and work stress in the present study did not influence markers of acute AKI, participants were less sensitive to increases in thermoregulatory and cardiovascular strain during a state of metabolic alkalosis.

## 1 INTRODUCTION

A large proportion of the world's workforce is exposed to prolonged, hazardous periods of heat stress, physical exertion, or in many instances, a combination of the two. Professions such as mining, farm work, construction, and wildland firefighting require a wide range of physical tasks, each imposing varying degrees of metabolic, ergonomic, and cognitive stress that can be exacerbated by nefarious environmental conditions. These risks are exacerbated by rising global temperatures, where an estimated one‐third of heat‐related deaths are now attributed to climate change (Sorensen & Hess, 2022). Whether it be due to the climate in the region or the physical nature of the vocation itself, the physiological strain imposed on the body can be dangerous, potentially impairing human productivity and performance, but also exacerbate pre‐existing clinical complications such as high blood pressure, obesity, and diabetes, particularly in elderly workers (Sorensen & Hess, 2022). One organ vulnerable to these stressors is the kidney, where numerous studies have documented the combined pathophysiological effect of prolonged heat and physical activity manifesting into acute kidney injury (AKI) (Houck et al., 2022; Li et al., 2022; Schlader et al., 2017). Indeed, repetitive AKI induced by such exposure has been linked to the development of chronic kidney disease, specifically chronic kidney disease of unknown origin (CKDu) (Chapman, Hess, et al., 2021; Glaser et al., 2016; Park et al., 2019; Wesseling et al., 2013). As yet, it is still unknown as to what the proportional influence of these stressors (e.g., heat and physical work) is in contributing to CKDu.

Although the pathophysiological effect of heat stress on kidney function has been known for some time, recent advances in the ability to detect biomarkers specific to AKI risk (e.g., tubular injury markers) have led to a more direct line of empirical investigation into the proportional influence of heat exposure and/or physical work, considering both duration and intensity (Bongers et al., 2017; Houck et al., 2022; Schlader et al., 2017). The multifaceted nature of this question, however, has required somewhat of a reductionist approach to date. For example, the physiological implications of heat exposure on humans vary widely and are influenced by factors such as fluid intake and/or restriction, acclimation and/or acclimatization, pre‐existing comorbidities, etc. Similarly, physical exertion imposes different levels of stress depending on the intensity and duration, as well as fuel and fluid availability, environmental stress, and the fitness state (trained or untrained) of an individual. Moreover, determining the clinical significance of AKI observed in controlled laboratory settings may not translate into “real‐world”, ecologically valid environments that better reflect actual working conditions (e.g., fluid availability, productivity incentives, and mandatory breaks) and which can also significantly influence the global physiological stress response.

Research exploring effective mitigation strategies to reduce the incidence of AKI has included heat acclimation (Haroutounian et al., 2021), hydration and precooling (Chapman, Johnson, et al., 2021), and modifications in physical work rates and durations (e.g., increasing rest periods or breaks (Glaser et al., 2020)). A novel, low‐cost, and easily implemented strategy that has not received much attention is sodium bicarbonate (SB) supplementation as a strategy to mitigate metabolic stress during physical exertion and potentially alleviate renal work load by lowering glomerular filtration demand, reducing bicarbonate reabsorption, and preservation of kidney microcirculation. SB supplementation, often used to offset the deleterious metabolic effects of intense exercise, has been widely researched in various capacities since the early 1900's. Briefly, the kidneys produce bicarbonate (HCO_3_^−^), which in turn tightly regulates acid–base balance in the body and ultimately provides a “first line of defense” during high rates of metabolic flux (e.g., exertional, high intensity work or exercise). Since the early days of discovery, providing supplemental bicarbonate in the form of SB has been shown to increase the blood's bicarbonate reserves, often resulting in ~20% acute and temporary increase in blood buffering capacity (Siegler et al., 2016). Although more commonly used in athletic scenarios to improve exercise performance, ingesting SB has also been shown to expand plasma volume (Siegler et al., 2022), as well as reduce markers of oxidative and inflammatory stress (Peart et al., 2013), which again may lead to a reduction in overall kidney stress. Further justification is provided by recent evidence in murine models during recurrent episodes of dehydration, where a reduction in markers of kidney damage has been observed after SB supplementation (Sanchez‐Lozada et al., 2018). As such, we have begun exploring the efficacy of SB supplementation in a variety of environmental conditions and physical stressors (Masoud et al., 2024).

This study was designed specifically to address the efficacy of this supplement and the multifaceted nature of AKI as it is influenced by heat stress and physical exertion in an ecologically valid environment. As such, the study was conducted outdoors in the Sonoran Desert in June, where average temps consistently sit at 100°F (~38°C) with low humidity (~15%). Additionally, the desert Southwest has a high percentage of outdoor workers, many of whom work in construction and typically work long shifts under a great degree of heat exposure throughout the summer months. Specifically, we exposed a cohort of non‐heat acclimated individuals to 2 h of outdoor, self‐paced work following NIOSH extreme heat guidelines. The work consisted of a series of workstations designed to mimic construction‐like tasks. Our primary objective was to investigate whether ingesting SB would mitigate markers of AKI. We hypothesized that (1) 2 h of self‐paced work in hot conditions would be sufficient to induce AKI, and that (2) SB supplementation would mitigate the severity of AKI.

## 2 MATERIALS AND METHODS

### 2.1 Participants

Fourteen young healthy men and women (six women) participated in the study (mean ± SD: age: 26 ± 3, body mass: 83.1 ± 26.2 kg, and height: 173.2 ± 13.6 cm). The following inclusion criteria were adopted for this study: (a) no contraindications to exercise as indicated by the Physical Activity Readiness Questionnaire (PAR‐Q+) and the Exercise Pre‐Participation Health Screening Questionnaire for Exercise Professionals by the American College of Sports Medicine, (b) not currently taking medicine or experiencing illness that may influence the cardiovascular system, (c) not taken nonsteroidal anti‐inflammatory drugs 1 week before enrollment, (d) age between 18 and 55 years old, (e) free of skeletal muscle injuries for greater than 3 months. Participants were also prescreened for ingestible core temperature pill contraindications (e.g., impairment of the gag reflex, a swallowing disorder, diseases or disorders of the esophagus, etc.). The study was approved by the Institutional Review Board of Arizona State University (STUDY00018980) and conducted following the Declaration of Helsinki (revision of 2013). All volunteers provided written informed consent before their participation.

### 2.2 General procedures

A counterbalanced, randomized, double‐blind cross‐over design was employed in this study with an initial consenting visit and familiarization session (~1 h) followed by two experimental trials, separated by 1 week and conducted at the same time of day. During the initial consenting visit, participants were familiarized with the exercise tasks and other measurement techniques included in the study. The two experimental trials required participants to rotate through a circuit of five working stations over a 2‐h period (explained in Section 2.3) to simulate common construction tasks. The work‐to‐rest ratio followed the NIOSH Criteria for a Recommended Standard, Occupational Exposure to Heat and Hot Environments guide for work intensity corresponding to a specific environmental heat stress. Participants worked in “intervention pairs” (SB or placebo (PLA)) to account for potential differences in environmental conditions across weeks. All procedures were replicated across the two experimental trials.

### 2.3 Experimental trials

**FIGURE 1:**
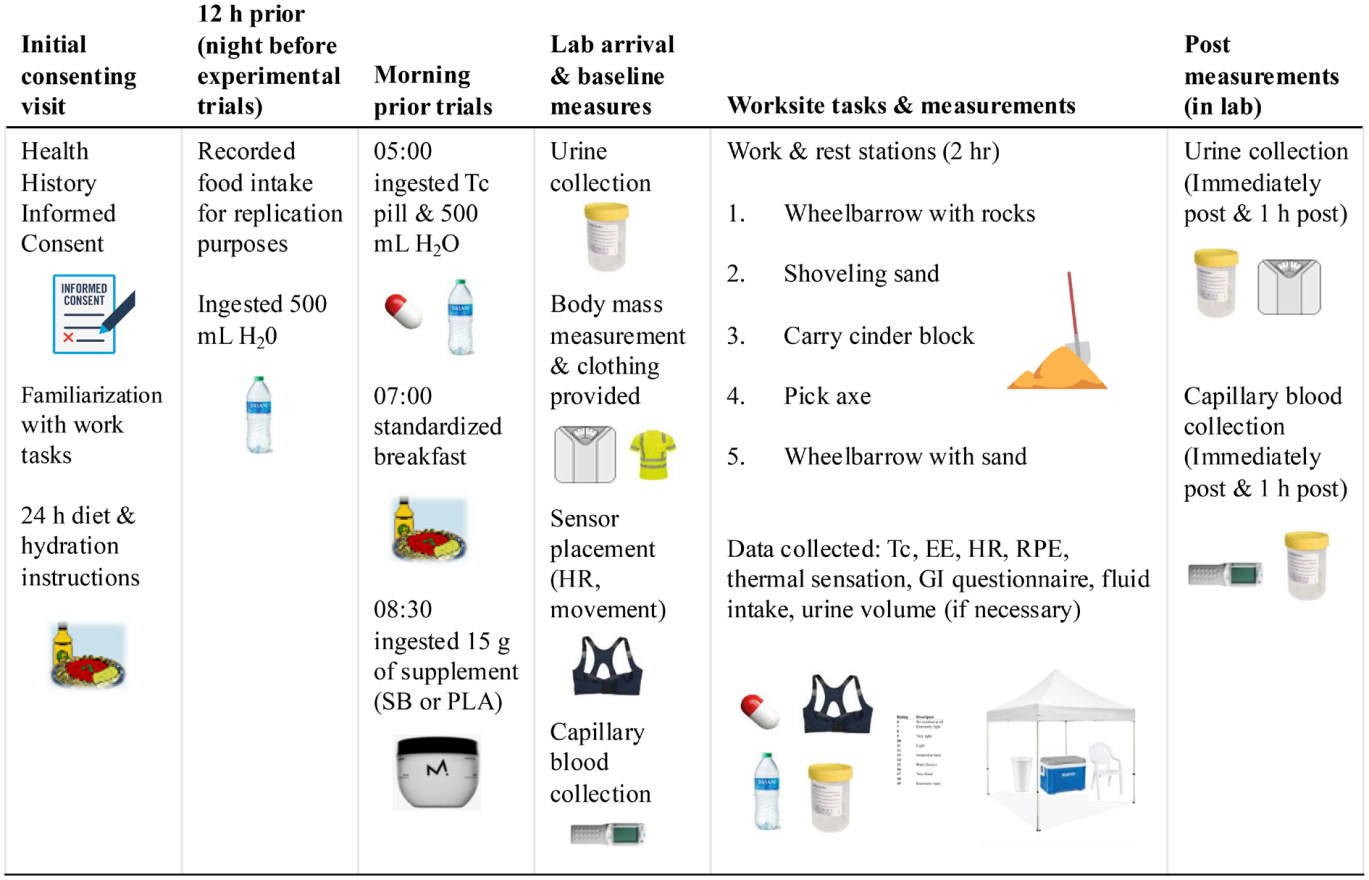
Study overview and flow diagram. Abbreviations are as follows: Core temperature (Tc), energy expenditure (EE), heart rate (HR), rating of perceived exertion (RPE), and gastrointestinal (GI).

A protocol schematic is provided in Figure 1. On the night and the morning before the experimental trial, participants recorded their food intake using a recall questionnaire provided by the research team and replicated the same diet for the subsequent trial. They also consumed 500 mL of water ~12 h prior to the experimental visit. At 05:00 on the morning of the experimental trial, they ingested a telemetric core temperature pill (e‐Celsius™) and an additional 500 mL of water. At 07:00, they consumed a standardized breakfast, and at 08:30 they consumed their supplement (SB or PLA) and recorded the time of their last urination before arriving to the lab at 09:00. Both sodium bicarbonate and the placebo were provided in‐kind by Maurten™ (Göteborg, SWE) and consumed as a standard 15 g amount incorporated within Maurten's patented hydrogel delivery system (containing 40 g of carbohydrate).

Upon arrival to the lab, participants voided their bladder in a urine container to measure urine volume, flow rate, and specific gravity (USG) to assess hydration status. Participants then measured their nude body mass (kg) using a digital scale (SECA, SECA 274, Hamburg, DEU), and subsequently dressed in work pants, a high‐vis long‐sleeve work shirt, gloves, and a sun hat (Harbor Freight, Phoenix, USA). Participants then sat quietly for approximately 10 min, when whole blood was collected from the fingertip into a heparinised 120 μL blood gas capillary tube and immediately analyzed for acid–base status (pH, bicarbonate (HCO_3_^−^) and base excess (BE)) (CG8+ cartridge, i‐STAT 1 Wireless, Abbott, Princeton, USA). The initial blood droplet was discarded, and the fingertip was gently massaged without applying excessive pressure to prevent sample contamination with interstitial fluid. Thereafter, blood samples were obtained upon completion of the work task (120 min), and 1 h post completion (Figure 1). Participants were also asked to rate any gastrointestinal (GI) stress using a questionnaire adapted with permission from Gaskell and colleagues (Gaskell et al., 2019). GI stress was again assessed at the midpoint of the work session, upon completion, and 1 h post. Lastly, participants were fitted with a Zephyr™ unit (Zephyr Performance Systems, Boulder, USA) for measurement of heart rate and to quantify physical activity.

Participants then walked outside to the mock job site to begin the physical work portion of the trial. The participants were assigned to one of five stations and rotated every 10 min for 1 h (Figure 1). The five construction work tasks were as follows: wheelbarrow with rock and sand (Stations 1 and 5): participants were asked to load the wheelbarrow with a designated number of rocks (~70 kg and 55 kg for men and women) and sand (~85 kg for men and 40 kg for women) and, once full, pushed the wheelbarrow for a distance of 20 m. Upon reaching the 20 m mark, the contents were dumped and the process was repeated for the duration of the station; shoveling (Station 2): participants were asked to shovel and move sand at a constant rate between piles 3 m apart using a shovel for the duration of the station; carrying (Station 3): participants were asked to carry one cinder block for a distance of 20 m, stopping and setting the cinder block down and picking it back up at 10 m increments for the duration of the station; picking (Station 4): participants were asked to pick the ground (dirt) using a 3 kg pickaxe within a square of 2 × 2 m for the duration of the station. Throughout all workstations, participants were instructed to work at a self‐paced intensity ranging from 11 to 13 on the Borg RPE scale (6–20; (Borg, 1970)).

As previously addressed, the work‐to‐rest ratio (W/R) followed the NIOSH Criteria for a Recommended Standard, Occupational Exposure to Heat and Hot Environments guide, where each 10 min workstation W/R was assessed via the current wet bulb globe temperature (WBGT) (Kestrel Heat Stress Tracker (5400 Kestrel Heat Stress Tracker, Lawrenceville, USA)). As per the guidelines for moderate intensity work, three WBGT limits were adopted: Green: 25–27.7°C, W/R—9/1 min; Yellow: 27.8–29.4°C, W/R—8/2 min; and Red: 29.5–31.6°C, W/R—5/5 min. During the rest breaks, participants were seated under a shaded tent, provided chilled water ad libitum (consumption volume recorded), and cold towels if desired. During this time, participants were also asked to provide a subjective rating of exertion (RPE) and rate their thermal sensation using a 10‐point scale (Wardenaar et al., 2023). A 10‐min break was provided between the two 1‐h work sessions. Once participants finished the work task and completed the final thermal sensation rating, they returned to the lab, voided their bladder, and measured their nude body mass to determine pre‐post changes. After a 10‐min seated rest, a second capillary sample was obtained, and the participants were instructed to remain sitting for 1 h, where final blood and urine samples were collected.

### 2.4 Methodological techniques and measurements (not addressed in experimental trials)

#### 2.4.1 Hydration status and urine flow rate

Urine samples for calculating urine volume and measuring USG were obtained before, during, and immediately after completing the work session to estimate glomerular filtration and assess hydration status. For each collection, participants were asked to capture all voided urine in graduated measuring containers. The volume recorded, along with the time from last urination, was used to estimate urine flow rate. Urine flow rate (expressed as mL.min^−1^) was calculated by dividing the volume of the urine sample collected by the time duration between previous urine void and the relevant collection time. Urine flow rate was used to normalize AKI markers (e.g., NGAL, etc.).

USG was measured in duplicate (samples averaged) using a handheld refractometer (Atago PALTMSeries, Tokyo, JPN). If the initial two measurements differed by more than 0.005, a third measurement was taken, and the median was considered for analysis.

#### 2.4.2 Heart rate and activity profile

Heart rate (HR) was measured continuously (1 Hz) during the work sessions using a sensor attached to a strap placed around the chest and lined up with the armpit (Zephyr Performance Systems, Boulder, USA). The activity level was measured using a triaxial accelerometer (band pass filtered and expressed in vector magnitude units (VMU) where x, y, and z are the averages of the three axial acceleration magnitudes over the previous second, and sampled at 100 Hz and accumulated for 1 s reporting), where walking is representative of ~0.2 VMU and jogging ~0.8 VMU (Zephyr Performance Systems, Boulder, USA). Data was averaged for each 10 min work station (including rest), with an aggregate calculated to represent cumulative work over the entire 2‐h period.

#### 2.4.3 Core body temperature

Core body temperature (T_C_) was continuously monitored throughout the work session using the telemetric ingestible core temperature pill (e‐Celsius Performance™, BodyCap, Hérouville Saint Clair, FRA). The participants were asked to swallow the pill at 05:00 the morning of both experimental trials. During the experimental trial, core temperature data was obtained every 15 s (eViewer Performance monitor, BodyCap, Hérouville Saint Clair, FRA). For analysis purposes, the data was averaged for each 10 min work station (including rest).

#### 2.4.4 Kidney injury markers

AKI risk was assessed using ELISA kits to measure concentrations of insulin‐like growth factor‐binding protein 7 (IGFBP7) (ELH‐IGFBPRP1‐2, RayBiotech Life, Peachtree Corners, GA; intra‐assay coefficient of variation [CV] = 5.5%), tissue inhibitor of metalloproteinase 2 (TIMP‐2) (ELH‐TIMP2‐2, RayBiotech Life, Peachtree Corners, GA; CV = 4.6%), and neutrophil gelatinase‐associated lipocalin (NGAL) (BPD‐KIT‐036, Enzo Life Sciences, Farmingdale, NY; CV = 6.6%), in urine pre and 1 h post work. Studies have reliably used 1 h post‐exercise urine sampling to detect transient changes in urinary AKI markers such as NGAL, TIMP2, and IGFBP‐7 (Bongers et al., 2017; Chapman et al., 2020; Houck et al., 2022). These time points are often used because they strike a balance between capturing peak excretion of injury markers and ensuring participant compliance, particularly in field settings. Urine samples were centrifuged before analysis, and resultant supernatant was used for analyses. Samples were diluted before analyses for IGFBP7 (1:50), TIMP‐2 (1:60), and NGAL (1:250) to ensure the obtained values were on the standard curve for each ELISA. The kidney markers were normalized to UFR, considering changes in urine production that may happen in dehydration conditions such as exercise in the heat. All ELISA analyses were completed according to manufacturer guidelines. All assays were analyzed in duplicate, and intra‐assay coefficient of variations for all markers was <10%.

### 2.5 Statistical analyses

Descriptive data are presented as mean ± SD with all statistical analyses being completed using IBM SPSS Statistics version 28 (SPSS Inc., Chicago, USA). Continuous variables were assessed for normality using the Shapiro–Wilk test, and variables with non‐normal distributions (IGFBP‐7 and NGAL) were log‐transformed (base‐10) to improve normality and stabilize variance. Changes in acid–base balance, thermotolerance and regulation, cardiovascular function, perceived effort and work achieved, and markers of kidney injury throughout the experimental trials were analyzed using a linear mixed model with two‐way repeated measures (condition × time). When significant time, condition, or interaction effects were detected, multiple post hoc pairwise comparisons were made using a Bonferroni procedure. Mean differences and standard error (SE) between conditions, as well as 95% confidence intervals (CI), were calculated when significant changes were observed. Two‐tailed statistical significance was accepted at an alpha level of <0.05.

## 3 RESULTS

### 3.1 Environmental work conditions

During data collection, the average temperatures within the 2‐week study period were 94.6°F (34.8°C) [min to max: 87.8°F (31°C) to 102.5°F (39.2°C)], while humidity averaged 18.8% (min to max: 11.2%–29.9%). Average temperatures and humidity's were not different across SB and PLA conditions (*p* > 0.84).

### 3.2 Blood acid–base balance, strong ions, and GI tolerance

**FIGURE 2:**
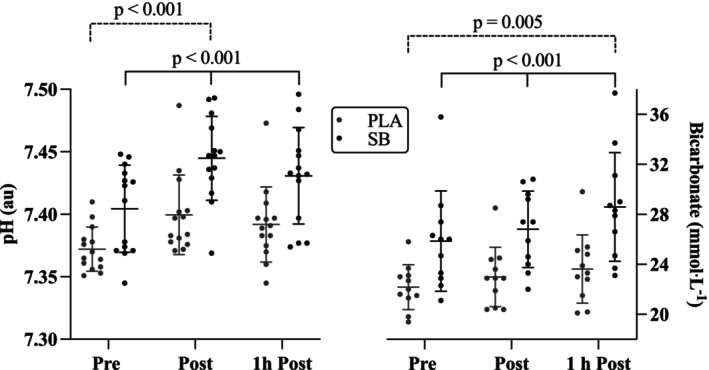
Individual and mean ± SD values for acid–base (pH (au) and blood bicarbonate (HCO_3_^−^; mmol·L^−1^)) prior to the 2‐h outdoor work session (Pre), immediately upon completing the session (Post) and again 1‐h after completion (1 h Post) for the two experimental sessions (sodium bicarbonate (SB) and placebo (PLA)). A main effect for condition (*p* < 0.001) and time (*p* < 0.001) indicated pH was elevated during the SB condition compared to PLA, and that both conditions were elevated from pre to post work. Similarly, bicarbonate was significantly elevated in the SB condition (*p* < 0.001) with a time effect evident from the start of work to 1 h post (*p* = 0.005).

Whole blood acid–base findings were consistent with induced states of metabolic alkalosis in the SB condition (Table 1, Figure 2). Significant condition (mean difference 0.39 ± 0.01 au (95% CI 0.05–0.03 au); *p* < 0.001) and time (mean difference pre to post 0.34 ± 0.01 au (95% CI 0.06–0.01 au); *p* < 0.001) effects were evident for blood pH. There was no significant interaction effect (condition × time) observed (*p* = 0.76). Similarly, SB resulted in a significant condition (mean difference 4.5 ± 0.7 mmol·L^−1^ (95% CI 5.8–3.2 mmol·L^−1^); *p* < 0.001) and time (mean difference pre to 1 h post 2.6 ± 0.8 mmol·L^−1^ (95% CI 4.6–0.6 mmol·L^−1^); *p* = 0.005) effects for blood bicarbonate (HCO_3_^−^) and base excess (BE) (condition: mean difference pre to post −4.5 ± 0.6 meq·L^−1^ (95% CI −5.6 to −3.3 meq·L^−1^); *p* < 0.001); time: mean difference pre to 1 h post −2.7 ± 0.7 meq·L^−1^ (95% CI −4.5 to −0.9 meq·L^−1^; *p* < 0.012). As with pH, there were no significant interactions for HCO_3_^−^ (*p* = 0.47) and BE (*p* = 0.62).

**TABLE 1:** Whole blood acid–base (pH, bicarbonate (HCO_3_^−^), base excess (BE)) and select ions (Na + and K+) for placebo (PLA) and sodium bicarbonate (SB) treatments from pre‐work (Pre), post‐work (Post), and 1 h post‐work (1 h Post). Data are presented as mean ± SD (95% CI).

	Pre	Post	1 h post
pH (au)			
PLA	7.37 ± 0.02 (7.36 to 7.39)	7.40 ± 0.03 (7.38 to 7.42)^b^	7.39 ± 0.03 (7.38 to 7.41)^b^
SB	7.40 ± 0.03 (7.39 to 7.42)^a^	7.45 ± 0.03 (7.43 to 7.46)^a^, ^b^	7.43 ± 0.04 (7.41 to 7.45)^a^, ^b^
HCO_3_^−^ (mmol·L^−1^)			
PLA	21.9 ± 1.8 (20.3 to 23.5)	22.9 ± 2.1 (21.3 to 24.5)	23.6 ± 2.4 (22.0 to 25.2)^b^
SB	25.7 ± 3.7 (24.1 to 27.3)^a^	27.0 ± 2.8 (25.4 to 28.6)^a^	29.2 ± 4.5 (27.6 to 30.9)^a^, ^b^
BE (meq·L^−1^)			
PLA	−3.4 ± 1.8 (−4.9 to −2.0)	−1.8 ± 2.5 (−3.2 to −0.3)^b^	−1.4 ± 2.7 (−2.9 to 0.0)^b^
SB	0.2 ± 2.9 (−1.2 to 1.7)^a^	2.9 ± 3.2 (1.5 to 4.4)^a^, ^b^	2.2 ± 3.4 (2.1 to 5.0)^a^, ^b^
Na^+^ (mmol·L^−1^)			
PLA	140.3 ± 1.6 (139.3 to 141.2)	138.1 ± 1.8 (137.1 to 139.0)^b^	139.0 ± 1.5 (138.0 to 139.9)^b^
SB	141.3 ± 1.7 (140.4 to 142.2)^a^	140.5 ± 2.0 (139.6 to 141.4)^a^, ^b^	140.1 ± 1.8 (139.2 to 141.1)^a^, ^b^
K^+^ (mmol·L^−1^)			
PLA	4.3 ± 0.3 (4.2 to 4.5)	4.4 ± 0.3 (4.2 to 4.5)	4.2 ± 0.3 (4.0 to 4.3)^b^
SB	4.2 ± 0.3 (4.0 to 4.3)^a^	4.0 ± 0.3 (3.9 to 4.2)^a^	3.9 ± 0.2 (3.7 to 4.0)^a^, ^b^

^a^ Main effect of condition (different from PLA): *p* < 0.001.

^b^ Main effect of time: *p* < 0.023; post hoc differences from Pre: *p* < 0.026.

The SB condition also resulted in a significant condition (mean increase in blood sodium (Na^+^) compared to PLA of 1.6 ± 0.4 mmol·L^−1^ (95% CI 2.3–0.8 mmol·L^−1^); *p* < 0.001) and time (mean increase pre to 1 h post 1.3 ± 0.5 mmol·L^−1^ (95% CI 0.1–2.4 mmol·L^−1^); *p* = 0.021) effect (Table 1). Blood potassium (K^+^) was also significantly lower in the SB condition (mean difference 0.3 ± 0.1 mmol·L^−1^ (95% CI 0.4–0.2 mmol·L^−1^); p < 0.001), while a main effect of time demonstrated a decrease from pre to 1 h post trial (mean decline of 0.2 ± 0.1 mmol·L^−1^ (95% CI 0.0–0.4 mmol·L^−1^); *p* = 0.023) (Table 1). No significant interaction effects were present for Na^+^ (*p* = 0.27) or K^+^ (*p* = 0.39).

There were no interactions (*p* = 0.96), conditions (*p* = 0.11), or time effects (*p* = 0.90) evident for GI tolerance between PLA and SB.

### 3.3 Thermal responses and hydration

**FIGURE 3:**
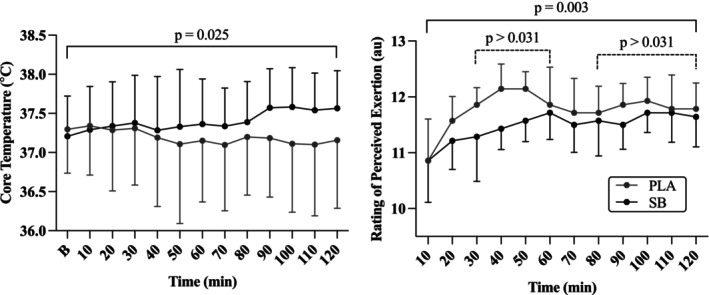
Mean ± SD values for core temperature (°C) and rating of perceived exertion (au) throughout the 2‐h outdoor work session for the two experimental sessions (sodium bicarbonate (SB) and placebo (PLA)). A main effect for condition indicated core temperature during the SB condition was higher than PLA (*p* = 0.025). For rating of perceived exertion, the PLA condition was higher than SB (*p* = 0.003), while both conditions were elevated when compared to the start of work (*p* > 0.31).

Core temperature was elevated during the work session in the SB compared to PLA (mean increase of 0.2 ± 0.1°C (95% CI 0.2–0.4°C); *p* = 0.025), but both SB and PLA remained steady throughout (*p* = 1.0) and there were no interaction effects (*p* = 0.99) (Table 2, Figure 3). Thermal sensation ratings were not different between conditions (*p* = 0.54), with a significant increase by 80 min from the start of work (mean increase of 1.1 ± 0.3 au (95% CI 0.1–2.5 au); *p* < 0.001; post hoc differences from the start of work to 80 min through to the end of the session: *p* < 0.038). There was no significant interaction effect observed (*p* = 1.0).

**TABLE 2:** Physiological responses at the start of work, at the mid‐point just prior to the 10 min break, and upon completing the final work station. Data are presented as mean ± SD (95% CI).

	End of 1st work station	Midpoint prior to 10 min break	End of final work station
Core temperature (°C)			
PLA	37.3 ± 0.6 (36.9–37.7)	37.2 ± 0.8 (36.7–37.6)	37.2 ± 0.7 (36.7–37.6)
SB	37.2 ± 0.5 (36.7–37.7)^a^	37.4 ± 0.6 (36.9–37.8)^a^	37.6 ± 0.5 (37.1–38.0)^a^
Heart rate (bpm)			
PLA	118 ± 20 (105–130)	110 ± 22 (97–122)^b^	113 ± 26 (101–126)^a^
SB	124 ± 18 (110–139)^a^	117 ± 31 (102–132)^a^, ^b^	124 ± 23 (109–139)^a^, ^b^
Activity level (VMU (g))			
PLA	0.26 ± 0.09 (0.21–0.31)	0.25 ± 0.11 (0.20–0.30)	0.28 ± 0.10 (0.22–0.32)
SB	0.31 ± 0.06 (0.25–0.37)	0.27 ± 0.12 (0.21–0.33)	0.25 ± 0.08 (0.20–0.31)
Rating of perceived exertion (RPE) (au)			
PLA^b^	10.9 ± 0.2 (10.4–11.3)	12.0 ± 1.2 (11.4–12.3)	12.0 ± 0.8 (11.3–12.3)
SB^a^, ^b^	11.0 ± 1.3 (10.4–11.3)	11.7 ± 0.8 (11.2–12.2)	11.6 ± 0.9 (11.2–12.1)
Thermal sensation score (au)			
PLA	2.6 ± 1.3 (2.0–3.2)	3.4 ± 1.0 (2.8–4.0)^b^	3.9 ± 1.4 (3.3–4.5)^b^
SB	2.6 ± 1.7 (2.0–3.2)	3.4 ± 1.0 (2.8–4.0)^b^	3.8 ± 1.1 (3.2–4.4)^b^

^a^ Main effect of condition (different from PLA): *p* < 0.025.

^b^ Main effect of time: *p* < 0.031; post hoc differences from the End of 1st Work Station: *p* < 0.038.

Body mass (kg) was not different between conditions at baseline (*p* = 0.85), did not change during the work session (*p* = 0.99) (Table 3), nor was there an interaction effect (*p* = 0.84). Participants were adequately hydrated in both conditions upon starting the work session (average USG 1.017 ± 0.002). USG remained stable throughout the work session across conditions (*p* = 0.72) and time (*p* = 0.70) (Table 3), and there were no interaction effects (*p* = 0.71). Fluid intake (SB: 1458 ± 630 mL; PLA: 1567 ± 643 mL; *p* = 0.35) and sweat rate were also not different between conditions (SB: 876 ± 298 mL·h^−1^; PLA: 815 ± 298 mL·h^−1^; *p* = 0.41).

**TABLE 3:** Hydration indices for pre‐work (Pre), post‐work (Post), and 1 h post‐work (1 h Post). Data are presented as mean ± SD (95% CI).

	Pre	Post	1 h post
Body mass (kg)			
PLA	82.2 ± 24.3 (69.4–94.3)	83.6 ± 23.8 (70.8–96.3)	
SB	82.3 ± 23.8 (69.5–95.0)	82.3 ± 22.9 (68.4–93.9)	N/A
Urine specific gravity (USG)			
PLA	1.018 ± 0.008 (1.013–1.023)	1.016 ± 0.009 (1.011–1.021)	1.014 ± 0.009 (1.010–1.019)
SB	1.016 ± 0.008 (1.012–1.021)	1.018 ± 0.007 (1.013–1.022)	1.016 ± 0.010 (1.012–1.021)

### 3.4 Physical activity and perceived exertion (RPE)

Average work time per station was not different between SB and PLA (SB: 7.8 ± 1.3 min worked per station; PLA: 7.8 ± 1.4 min worked per station; *p* = 0.87). Activity level, as quantified by VMU (g), was not different between conditions (*p* = 0.69), stayed constant throughout the work session (*p* = 0.96) (Table 2), and there were no interaction effects (*p* = 0.74). Heart rate (HR) was consistently higher in the SB trial (mean difference of 9 ± 3 bpm (95% CI 3–14 bpm); *p* = 0.002) than in PLA. Although a main effect of time was evident (*p* < 0.001), this was due to the increased HR in both conditions after the 10 min work break at the 1‐h mark. Interestingly, RPE was consistently higher in PLA compared to SB (mean increase of 0.3 ± 0.1 au (95% CI 0.1–0.5 au); *p* = 0.003), while both conditions steadily increased from 30 min through to the end of the work session (120 min) (mean increase of 1.0 ± 0.2 au (95% CI 0.2–1.8 au); *p* = 0.005; post hoc differences from the start of work to 30 min through to the end of the session: *p* < 0.031) (Figure 3). There were no interaction effects observed (*p* = 0.94).

### 3.5 Markers of kidney injury

**FIGURE 4:**
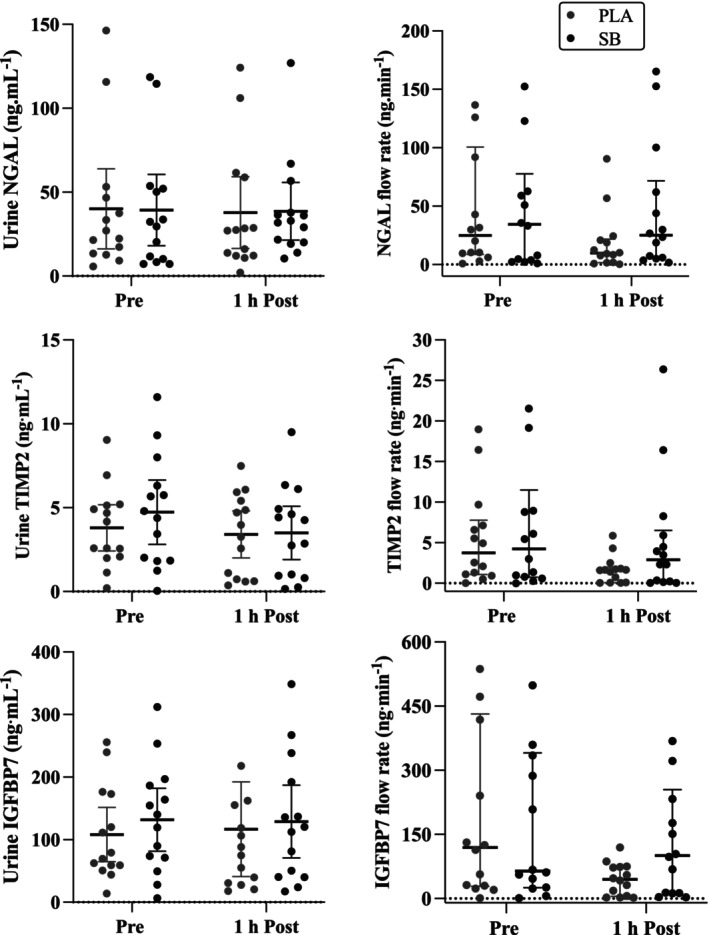
Individual and mean ± SD values for urinary neutrophil gelatinase‐associated lipocalin (NGAL), tissue inhibitor of metalloproteinase 2 (TIMP2), and insulin‐like growth factor‐binding protein 7 (IGFBP‐7) concentrations (both raw and corrected for urine flow rate (ng·min^−1^)) prior to the 2‐h outdoor work session (Pre) and again 1 h after completion (1 h Post) for the two experimental sessions (sodium bicarbonate (SB) and placebo (PLA)).

Urinary NGAL, TIMP2, and IGFBP‐7 concentrations (both raw and corrected for UFR) are presented in Figure 4 and Table 4. TIMP2 data was normally distributed as determined using the Shapiro–Wilk test for normality (*p* > 0.05) and used for analyses; however, IGFBP‐7 and NGAL were not normally distributed (*p* < 0.01) and thus log‐transformed prior to any comparisons. There were no statistically meaningful changes across the three markers from the start to 1 h post‐work session (Table 4; *p* > 0.27), no differences were evident between conditions (Table 4; *p* > 0.49), and no interaction effects (*p* > 0.49). UFR was also not different across conditions (PLA: 3.3 ± 0.5 mL·min^−1^; SB: 2.1 ± 0.5 mL·min^−1^; *p* = 0.13), did not change across the work session (*p* = 0.17), and had no interaction effect (*p* = 0.26).

**TABLE 4:** Urinary concentrations (raw data) for all kidney injury markers (NGAL, TIMP2, and IGFBP‐7) for pre‐work (Pre) and 1 h post‐work (1 h Post). Data are presented as mean ± SD.

	Pre	1 h post
NGAL (ng·mL^−1^)		
PLA	40.1 ± 41.4	37.8 ± 37.1
SB	39.3 ± 36.8	38.6 ± 29.8
TIMP2 (ng·mL^−1^)		
PLA	3.8 ± 2.4	3.4 ± 2.4
SB	4.7 ± 3.3	3.5 ± 3.1
IGFBP‐7 (ng·mL^−1^)		
PLA	108.2 ± 75.2	116.8 ± 131.0
SB	131.8 ± 87.0	129.0 ± 100.5

## 4 DISCUSSION

This study investigated the efficacy of sodium bicarbonate supplementation in mitigating markers of AKI during 2 h of outdoor, self‐paced work activity in the heat. Our observations did not support the hypothesis of SB supplementation reducing the incidence of AKI in this environment, as there were no meaningful differences or changes from pre‐ to post‐work in urinary NGAL, TIMP2, or IGFBP‐7 concentrations despite inducing a significant state of metabolic alkalosis in the SB condition. Notwithstanding maintaining a consistent level of fluid intake and hydration across both conditions, Tc and HR were significantly elevated in the SB condition, but perception of effort (RPE) was lower when compared to PLA. Although no differences in work activity were evident, as quantified by VMU, this may suggest participants were less sensitive to increases in thermoregulatory and cardiovascular strain during a state of significant metabolic alkalosis.

A critical determinant of AKI risk is the magnitude of hyperthermia (passive or active) and the availability of fluid (Chapman et al., 2020). Chapman and colleagues have empirically demonstrated the deleterious effects of both hyperthermia and dehydration on the risk of AKI, observing increased concentrations of urinary NGAL and IGFBP7 during uncompensable heat exposure and fluid restriction (Chapman et al., 2020). However, others have observed comparable levels of AKI during prolonged, intermittent bouts of high‐intensity physical activity, or muscle damaging exercise in the heat, when adequate fluid was provided (Houck et al., 2022; Li et al., 2022). Another challenge is determining the clinical significance of AKI observed in controlled laboratory settings compared to more ecological environments that better reflect the state and physical condition of workers. Factors such as sociocultural and socioeconomical situations related to workplace conditions (e.g., fluid availability, productivity incentives, and mandatory breaks) can strongly influence global physiological stress. As such, we designed this study to purposely address whether or not SB supplementation would attenuate any incidence of AKI during work conditions adhering to NIOSH extreme heat guidelines. As a result, despite similar environmental conditions to previous studies (Bongers et al., 2017; Houck et al., 2022; Li et al., 2022; Schlader et al., 2017), the implementation of standardized work‐to‐rest cycles, shade, and hydration protocols prevented the incidence of AKI regardless of the intervention (Figure 4).

Direct comparisons between our findings and lab‐based studies are challenging due to differences in work rates, durations, and levels of environmental heat stress, with many studies reporting significant increases in AKI markers following heat exposure involving higher‐intensity workloads or longer exposure durations (Houck et al., 2022; Li et al., 2022; Schlader et al., 2017). The construction activities and work rates (e.g., 11–13 RPE) prescribed in the present study were implemented based on our pilot data collected outdoors using indirect calorimetry, as well as previous literature suggesting the energy cost of general construction activities to be ~5 kcal/min (or metabolic equivalents (MET) levels between 3 and 6 METS) (Poulianiti et al., 2019). Although our participants were exposed to persistent solar radiation and high ambient temperatures (~35°C–39°C), short and frequent breaks, ad libitum fluid intake, and moderate work rates provided effective protection from thermoregulatory and cardiovascular strain than previously observed in lab‐based simulations (Morrissey‐Basler et al., 2024). In a much longer temperate exposure (~13 to 24°C; ~8 h) at a similar intensity range to the present study (e.g., HR ~ 112 bpm), Bongers and colleagues observed a two‐fold increase in urinary NGAL post‐work, suggesting a potential duration effect even at lower intensities regardless of ambient temperatures (Bongers et al., 2017). However, as the authors acknowledge, dehydration may also have influenced kidney function, as ad libitum fluid intake resulted in 1%–2% loss in body mass over the exercise duration. Given that our participants remained hydrated and followed a regulated work‐rest schedule, the physiological strain in our study may have been insufficient to provoke a meaningful AKI response.

Although redundant due to the heat mitigation strategies in the present study, there are a number of plausible mechanisms in which SB supplementation may play a complementary role in reducing AKI. The challenge in determining to what extent, if any, SB may mitigate AKI again lies in the multi‐faceted heat tolerance/exercise intensity/exercise duration paradigm. Exercise intensity most likely is a primary player, as both short and prolonged high‐intensity exercise accelerate metabolite production that can be acutely supported through SB supplementation (e.g., increased H^+^ buffering). As such, it is plausible that SB supplementation may also reduce H^+^ excretion rates and HCO_3_^−^ reabsorption, thereby lessening the compensatory burden on the kidneys. The increase in cellular and oxidative stress commonly observed during these tasks has also been shown to be reduced after SB ingestion. During single (4‐min “all out” effort) and multiple, repeated sprints (10 × 15 s @ 120% peak power), Peart and colleagues observed attenuated heat shock protein (HSP 72—a marker of cellular heat stress) and lipid peroxidation (thiobarbituric acid reactive substances or TBARS) after supplementing with 0.3 g·kgBW^−1^ SB 90 min prior to exercise (Peart et al., 2011, 2013). The increased lipid peroxidation caused by myoglobin (Mb) released during muscle damaging exercise has also been shown to be attenuated during alkalinization, potentially reducing the reactive oxygen species (ROS)‐induced oxidative damage (Moore et al., 1998).

As work intensity in the present study was restricted to an RPE of 12 to 13 in order to more closely mimic construction activities (Poulianiti et al., 2019), and even with the 2 h duration, exercise‐induced cellular stress most likely would have been minimal and therefore limited the efficacy of SB in this instance. However, establishing a “work‐intensity threshold” may be warranted given our recent findings demonstrating an alkalosis‐induced reduction in markers of AKI during moderate intensity exercise (Masoud et al., 2024). Even at low intensities, however, prolonged heat exposure can increase ROS production in the body particularly in heat naïve individuals. As the participants in the present study were not outdoor workers, we hypothesized that the prolonged heat exposure coupled with the unaccustomed working tasks would be enough to induce a level of physiological strain where the addition of SB would result in a differential effect on markers of kidney stress. Interestingly, even with the self‐paced directive, we observed a small but consistent decrease in RPE but elevated HR and Tc, suggesting a greater physiological strain was present in the SB condition (Table 2, Figure 3). The increase in Tc and HR, coupled with the reduced perception of effort in the SB condition, may suggest a somewhat blunted level of afferent feedback which we have observed in previous studies (Siegler & Marshal, 2015). Katagiri and colleagues (Katagiri et al., 2021) observed a similar reduction in RPE after SB supplementation during 60 min of cycling in 35°C (40% humidity) at a moderate, steady‐state intensity (50% VO_2_max), which the authors attributed to an alkalosis‐induced attenuation in cerebral hypoperfusion caused by hyperthermia‐induced hyperventilation, ultimately alleviating central fatigue during exercise in the heat. These authors did not observe an increase in core temperature in the SB condition, however, possibly due to the fixed intensity and/or shorter duration of the task (Katagiri et al., 2021). Ultimately, and regardless of the increased cardiovascular strain, the magnitude of change was not enough to manifest into meaningful differences in the post markers of AKI.

The primary limitation of the present study stemmed from the low physiological strain due to the implementation of the NIOSH Criteria for a Recommended Standard: Occupational Exposure to Heat and Hot Environments. The criteria effectively mitigated the heat stress, and the prescribed work rate (~11–13 RPE) did not elevate Tc to levels previously shown to correlate with markers of kidney injury (Houck et al., 2022; Li et al., 2022). However, we felt it important to investigate the practical efficacy of this supplement in a more ecologically valid environment in order to better contextualize when this intervention may be appropriate, specifically in light of our recent findings supporting SB supplementation during slightly higher environmental stress and exercise loads (Masoud et al., 2024). A longer work session (e.g., 8–10 h) or increased workload intensities may have induced a more pronounced increase in markers of AKI (Bongers et al., 2017), perhaps increasing the possibility of exertional muscle damage and creating conditions where SB supplementation might exert a protective role. Additionally, future studies should incorporate longitudinal assessments with additional markers (e.g., serum creatine kinase, serum creatinine, etc.), as chronic SB supplementation may have cumulative protection in the kidneys over repeated work bouts.

In conclusion, 2 h of outdoor work in the heat, following NIOSH criteria, provided protection from AKI as indicated by no changes in urinary NGAL, TIMP2, or IGFBP‐7 concentrations. As a consequence, the metabolic stress imposed by the physical work did not sufficiently induce stress on renal function, and therefore we are unable to determine whether SB supplementation is an effective mitigation strategy under these circumstances. In these conditions, however, participants in the SB condition exhibited a lower RPE despite an elevated HR and Tc, suggesting potential alterations in perceptual responses to physiological strain. Future research should investigate SB supplementation under higher‐intensity workloads and extended exposure durations, where renal stress is more pronounced, and SB's potential protective effects can be more rigorously evaluated.

## AUTHOR CONTRIBUTIONS

JS and FA conceived and designed research. JS, BB, and RF performed the experiments and analyzed the data. All authors were involved in interpreting the results, preparing figures, drafting and editing the manuscript, and have approved the final version.

## FUNDING INFORMATION

CHS Heat and Health Pilot (internal support within the College of Health Solutions, Arizona State University).

## DATA AVAILABILTY STATEMENT

Raw data is available upon request.

## ETHICS STATEMENT

The study was approved by the Institutional Review Board of Arizona State University (STUDY00018980) and conducted following the Declaration of Helsinki (revision of 2013). All volunteers provided written informed consent before their participation.
